# Association between Migraine and Workplace Social Support in the Social Context of China: Using a Validated Chinese Version of the DCSQ

**DOI:** 10.3390/healthcare11020171

**Published:** 2023-01-05

**Authors:** Du Wei, Yue Chang, Xiaolong Lu, Xingying Fan, Jiaqi Hu, Otilia Manta, Mohammed K. A. Kaabar

**Affiliations:** 1 School of Medicine and Health Management, Guizhou Medical University, Guiyang 550025, China; 2 Department of Social and Preventive Medicine, Faculty of Medicine, Universiti Malaya, Kuala Lumpur 50603, Malaysia; 3 Center for Financial and Monetary Research “Victor Slavescu”, Romanian Academy, 012101 Bucharest, Romania; 4 The Research Department, Romanian-American University, 012101 Bucharest, Romania; 5 Institute of Mathematical Sciences, Faculty of Science, Universiti Malaya, Kuala Lumpur 50603, Malaysia

**Keywords:** migraine disorder, social support, demand-control-support questionnaire, reliability, validity

## Abstract

Background: Workplace social support might have a protective function against migraine in the social context of China, as close co-worker relationships and collectivism are acknowledged as work values in Chinese society. Objectives: This paper aimed to analyse the association between migraine and workplace social support. The validity and reliability of the Chinese version of the Support scale of the Demand-Control-Support Questionnaire (DCSQ) used were also determined. Methods: Following international guidelines, this study was carried out in two stages. Stage I involved translations and pilot testing to assess content and face validity of the Chinese version of the DCSQ Support scale. Stage II was a cross-sectional survey (N = 677 bank employees) to evaluate structural validity, internal consistency and test-retest reliability of the Support scale, as well as to examine the association between workplace social support and a migraine-positive diagnosis. Results: A high level of social support in the workplace was associated with a 74% decreased likelihood of migraine (adjusted OR = 0.26, 95%CI: 0.14–0.46). Of the six aspects of workplace social support, co-worker support had the greatest protective effect (adjusted OR = 0.49, 95% CI: 0.39–0.60). The Chinese version of the DCSQ Support scale established satisfactory content and face validity (I-CVIs ≥ 0.78; S-CVI_AVE_ ≥ 0.90). Confirmatory factor analysis verified its one-dimensional theoretical factor, with adequate internal consistency (Cronbach’s α 0.98; item-total correlations ≥ 0.80) and test-retest reliability (weighted Kappa coefficients 0.81–0.87; percentages agreement 85.23–88.92%). Conclusions: In the Chinese social context, workplace social support could protect against migraine, with the strongest benefit coming from co-workers. This study also provides a Chinese-language DCSQ Support scale as a valid and reliable instrument for measuring workplace social support.

## 1. Introduction

Migraine affects one out of every ten people worldwide, having an impact on individuals, employers and society [1]. This disease causes the second-greatest disability worldwide [2], and leads to a 40% loss in productivity [3]. Migraine is increasingly recognised as a public health concern [4]. Recent reviews have pointed out that migraine increases the risk of stroke and cardiovascular diseases [5,6]. Thus, according to the World Health Organisation (WHO) it is crucial to study protective factors for better migraine management [7].

An integrated review based on human and animal neurobiological studies concluded that social support could help protect against neurological dysfunction [8]. Social support is defined as the availability of assistance from others in times of need or crisis, including emotional, instrumental, informational and companionship support [9,10]. The impact of stronger social support on lower disease morbidity and mortality is via psychological, behavioural, and biological pathways [11].

Western epidemiological studies have often focused on the protective function of an important source of social support against migraine, workplace social support [12,13]. This is because global competition and rapid technological advancement have made work the centre of life [14,15]. It is necessary to investigate the effect of workplace social support in various countries, since it is likely to vary depending on culture and social settings [16]. In Chinese society, close co-worker relationships and collectivism are acknowledged as work values [17]; however, there is a scarcity of evidence on the association between workplace social support and migraine in the social context of China. Examining this association is of considerable importance in China, where migraines account for 151,600 (95% UI: 139,966–163,357) disability-adjusted life years each year [18]. Understanding the protective role of workplace social support against migraine in this context helps close existing knowledge gaps and highlight opportunities for migraine-related workplace interventions.

The Demand-Control-Support Questionnaire (DCSQ) was developed for research purposes and is based on Karasek’s job demand-control model [19], which is an instrument intended to be used among employees to inspect workplace psychosocial exposures related to health outcomes [20]. Furthermore, the Support subscale of the DCSQ is recommended for separate analysis from the Demand and Control subscales because previous research has demonstrated its distinct role compared to the other two subscales [21,22]. The DCSQ was originally developed in Swedish [20] and has since been validated in English [23], German [23], Spanish [24], Brazilian [22], Japanese [25] and other languages, with satisfactory validity and reliability. However, it has not yet been translated into Chinese.

The purpose of this research was to examine the association between migraine and workplace social support in the social context of China using the DCSQ. We hypothesised that individuals with high workplace social support would be less likely to suffer from migraine. In this study, we first translated the Support scale of the DCSQ into the Chinese language and assessed its validity and reliability.

## 2. Materials and Methods

This work was carried out in two stages. Stage I involved translations and pilot testing to determine content and face validity. Stage II was a cross-sectional survey to evaluate the structural validity, internal consistency, and test-retest reliability of the Support scale of the DCSQ, as well as to examine the association between workplace social support and migraine.

The COnsesus-based Standards for the Selection of Health Measurements Instruments (COSMIN) [26,27] and Sousa and Rojjanasrirat’s guidelines [28] were followed for the validity and reliability of the DCSQ Support scale, and the Reporting of Observational studies in Epidemiology (STROBE) guidelines [29] were followed for the association between migraine and social support in the workplace.

### 2.1. Instruments

Permission to use the following instruments was obtained from Dr. Töres Theorell and Dr. Timothy J Steiner, respectively.

#### 2.1.1. The Support Scale of DCSQ

The Support scale of the DCSQ is a six-item measure of workplace social support perception. Respondents pick each item on a four-point Likert response scale (1 = never, 2 = seldom, 3 = frequently and 4 = always). The total scores range from 6 to 24. According to the original study [20], we categorised workplace social support as low (a score of 6 to 12), medium (13 to 18) or high (19 to 24) levels. Before applying it to the Chinese context, we translated it into Chinese and tested its validity and reliability.

#### 2.1.2. The HARDSHIP Questionnaire

The HARDSHIP questionnaire, which was developed based on version three of the International Classification of Headache Disorders, was utilised for migraine diagnosis in this study [30]. The Chinese version of the HARDSHIP questionnaire has been translated and cross-culturally validated, with sensitivity and specificity being greater than 70% [31].

### 2.2. Stage I: Translations and Pilot Testing

#### 2.2.1. Translations

The translation process involved forward and backward translations, as detailed in Figure S1. To begin, two independent translators with knowledge of psychosociology and cross-cultural experience, whose mother tongue is Chinese, conducted the translations from English to Chinese. A third translator compared and summarised the discrepancies between the two forward translations. Any contradictions or ambiguities were addressed by a multidisciplinary committee comprised of the translators and members of our research team. Subsequently, two backward translations were completed independently by two translators with backgrounds as previously described and whose mother tongue is English. They were entirely ignorant of the original English version of the DCSQ. The two back-translated versions were compared with the original English version by the multidisciplinary committee. A consensus was achieved to produce a pre-final Chinese version of the Support scale.

#### 2.2.2. Pilot Testing

To determine content validity, a panel of seven experts (three public health specialists, one occupational health specialist, two sociologists and one expert with human resource management practice, all with a Ph. D degree or senior job title in relevant areas) were invited to evaluate the pre-final Chinese scale’s conceptual equivalence of ‘workplace social support’ and its aspects, using a four-point Likert scale ranging from ‘highly relevant and thorough’ (score 4) to ‘not relevant and not thorough’ (score 1). The I-CVIs and S-CVI_AVE_ were calculated as expert agreement [28,32]. Content validity is considered satisfactory when the I-CVIs and S-CVI_AVE_ are above 0.78 and 0.90, respectively [32]. The panel was then asked to assess whether any critical issues had been ignored and was encouraged to provide comments. All comments were scrutinised and analysed by our research teams.

Based on the suggested sample size of more than 50 to ensure face validity [33], 58 pilot samples were recruited from the DCSQ’s target population—the employed population—through purposive sampling, which ensured participant variation. Their language is Chinese. Participants were asked to evaluate the clarity of the instructions, response format and all items on the Chinese version of the DCSQ Support scale using a dichotomous question (clear/unclear) [28]. Following each question, there was a section where participants could make comments or rewrite sentences to help make this Chinese version of the Support scale easier to understand by the target population.

### 2.3. Stage II: A Cross-Sectional Survey

#### 2.3.1. Participant Recruitment

A cross-sectional survey was performed among employees in the banking sector in Guizhou province in China from May 2022 to September 2022. Bank employees appear to be at increased risk for migraine, with a prevalence rate as high as 47% [3,34], and established occupational risk factors such as sedentary desk work [35], forward head postures [36], repetitive tasks [35], air conditioning systems [37] and high-stress levels [38].

Chinese citizens between the ages of 18 and 60, who had been full-time bank employees for at least 6 months, fulfilled the inclusion criteria, whereas part-time employees were excluded. Bujang et al. proposed a sample size formula of n = 100 + 50i, where ‘i’ denoted the number of predictors for logistic regression [39], and, in addition, a minimum of 300 cases is required to ensure proper convergence of estimates when dealing with ordinal data for confirmatory factor analysis [40]; therefore, considering the above, a sample of over 600 bank employees was accessed at this stage.

Participants were chosen from the two largest public banks in China, the Industrial and Commercial Bank of China and the China Construction Bank using two-stage probability sampling. The Primary Sampling Units were the bank branches in Guizhou province, obtained from *Qichacha*, a database of Chinese corporate records. Because of resource constraints, 14 bank branches were selected through a probability proportional to size method. The full-time employee lists of the selected bank branches served as the Second Sampling Units. We attempted to randomly choose the same proportion of employees from each bank branch, but due to disparities in subject accessibility, we received various response proportions from different selected bank branches. To ensure an equal probability of the sample, a weighting procedure was applied. Each respondent was given a weight equal to the reciprocal of the probability of being selected.

Trained interviewers administered the investigation twice at the selected banks, with a suggested time interval of one to two weeks [41]. Face-to-face interviews performed in the same way helped clarify important questions, eliminating information bias. The first survey comprised the final version of the DCSQ Support scale, the HARDSHIP questionnaire and socio-demographic details (age, sex, education, occupation, location of work, relationship status and monthly salary), and the second survey asked them to complete the DCSQ Support scale again. During both surveys, no noteworthy events occurred.

#### 2.3.2. Statistical Analysis

Using data from the cross-sectional survey, the Support scale was tested for structural validity, internal consistency and test-retest reliability. Furthermore, the associations between social support in the workplace and migraine diagnosis were examined.

To determine whether the Chinese version of the DCSQ Support scale synchronised the same one-dimensionality as the original scale, confirmatory factor analysis (CFA) was conducted on a one-factor model. To avoid bias in the parameter estimates, univariate and multivariate normality were verified first [42]. Univariate normality was tested by the absolute values of skewness (<2) and kurtosis (<7), as common normality tests, such as the Shapiro-Wilk or Kolmogorov-Smirnov tests, are too stringent when a sample size exceeds 300 [43]. Multivariate normality was tested by the Mardia’s test. The descriptive analyses of the six items of the DCSQ Support scale are shown in Table S1. In spite of the absolute skewness and kurtosis values below the thresholds for univariate normality, the *p*-value for the Mardia’s test revealed the violation of multivariate normality. Given the ordinal and non-normal Likert data, the weighted least squares mean and variance adjusted estimator (WLSMV) [44,45] was used in the CFA model. The following indices were calculated to assess the model’s overall goodness of fit: [46,47] (1) root mean square error of approximation (RMSEA) < 0.06; (2) SRMR < 0.06; (3) comparative fit index (CFI) and Tucker-Lewis index (TLI) > 0.95. The *Lavaan* package in R was used for the CFA. The internal consistency was measured by Cronbach’s α and item-total correlation. When the value of Cronbach’s α is ≥0.7 and the values of item-total correlation are ≥0.3, internal consistency is deemed high [46,48]. Weighted kappa and percentage agreement were computed to assess the test-retest reliability of the Likert ordinal scores. A weighted kappa coefficient ≥0.7 is regarded to indicate excellent stability over time [46].

All respondents were classified as migraine positive or migraine negative for the diagnosis over a one-year time frame of prevalence, based on the HARDSHIP’s algorithm. The weighted number of cases and weighted percentages were used to describe the categorical variables. Chi-square tests (or Fisher’s exact tests when needed) were used for univariate analyses. Multivariable logistic regression models were performed to examine factors influencing a positive diagnosis of migraine, in which the three categories of workplace social support served as independent variables. We ran two unadjusted and adjusted models with socio-demographic covariates as confounders. In addition, to analyse the effect of each aspect of workplace social support, we conducted separate sub-analyses, with each aspect being an independent variable independently. Before running these models, we checked whether the data conformed to the assumptions of logistic regression. Linearity was checked through the Box-Tidwell procedure [49]. The *Logistf* package in R, which performed the penalised maximum likelihood estimation (PMLE), was used for the logistic regression because of the sparse data commonly observed in prevalence studies. The significance level was set at a *p*-value of 0.05.

## 3. Results

### 3.1. Socio-Demographic Characteristics and Migraine Diagnosis

A total of 693 bank workers were confirmed to be eligible. Multiple endeavours were embarked upon to approach those who did not respond the first time. Of them, 16 subjects refused to participate. The remaining 677 respondents had no missing data and were thus included in the analysis.

Table 1 displays their socio-demographic characteristics. The sample consisted of 258 males (38.07%) and 419 females (61.93%). Their ages ranged from 18 to 60 years old. The majority worked as cashiers and loan officers (72.67%), were married or in a relationship (75.48%), worked in urban areas (95.72%) and held a bachelor’s degree or higher (82.27%). Regarding social support in the workplace, 63.96% reported a high level, 20.24% a medium level and 15.80% a low level.

As shown in Table 1, 23.63% (160/677) of bank employees were diagnosed with migraine. Migraine participants differed from non-migraine participants in most socio-demographic factors and workplace social support. In comparison to those with high social support, those with low and medium workplace social support had a higher migraine prevalence (42.99% and 40.88% vs. 13.39%, *p* = 0.000).

### 3.2. Content Validity and Face Validity

The expert panel evaluated the content of the pre-final Chinese version of the Support scale of the DCSQ. The content of Item 1, “There is a calm and pleasant atmosphere where I work” was changed to “There is a cohesive atmosphere where I work”, and the content of Item 4, “The others understand if I have a bad day” was changed to “The others are ready to hear my demands”. These modifications were made to accommodate the local culture and language. The meaning of both items, however, remained unchanged from the original. All of the panellists agreed on their relevance and comprehensiveness. Our research team modified these expressions in accordance with the suggestions of the panel. Consequently, all I-CVIs and S-CVI_AVE_ were above the cut-off points of 0.78 and 0.90, respectively.

When it came to face validity, all pilot participants reached a consensus that the concept of ‘workplace social support’ and all the critical components covered by the Chinese version of the Support scale were adequately captured and that they were able to understand the instructions and the meaning of all items. Following that, the final Chinese version of the DCSQ Support scale was produced.

### 3.3. Structural Validity

**Figure 1:**
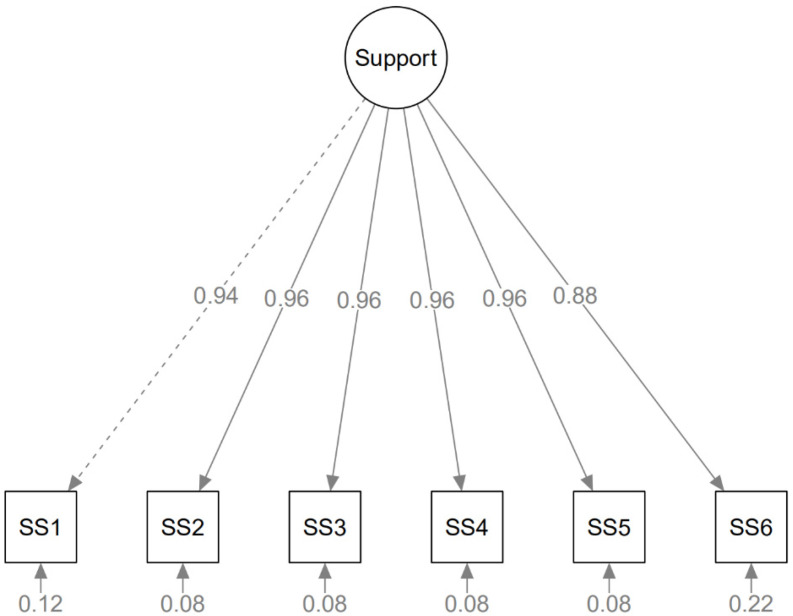
Path diagram of the DCSQ Support scale CFA model.

As described in the theory, a one-factor Support model was conducted. Figure 1 illustrates the CFA results for this model. It exhibited an adequate fit to the factor structure: RMSEA = 0.014 (95% CI: 0.000–0.047), SRMR = 0.008, CFI = 0.999, TLI = 0.998, standardised item-factor loadings ranged from 0.881 to 0.961.

### 3.4. Internal Consistency

The internal consistency of the Chinese version of the DCSQ Support scale was adequate, with a Cronbach’s α coefficient of 0.98. All corrected item-total correlation coefficients were >0.80.

### 3.5. Test-Retest Reliability

Table 2 shows the weighted Kappa coefficients and percentage agreement for the six items of the Support scale of the DCSQ. All items exhibited excellent agreement, with the weighted Kappa coefficients ranging from 0.81 to 0.87 and the percentage agreement ranging from 85.23% to 88.92%.

### 3.6. Associations between Migraine and Workplace Social Support

The assumptions of the binary logistic regression were tested and not violated. The results before and after adjusting for socio-demographic confounders are shown in Table 3. Having a medium level of social support in the workplace was not significantly associated with migraine risk; however, having a high level of social support was discovered to be associated with a 74% reduction in the odds of experiencing migraine (adjusted OR = 0.26, 95% CI: 0.14–0.46), although this association was reduced when confounders were controlled for.

To examine which aspect is the most influential, associations between the six aspects of workplace social support and a positive diagnosis of migraine are displayed in Table 3. The results indicated that all aspects (atmosphere, spirit of unity, co-worker support, co-worker help, relationship with supervisors and relationship with co-workers) were each significantly associated with a decreased risk of migraine. After controlling for gender, age, education, relationship, location of work, job and salary, a one-score increase in any aspect of social support in the workplace reduced the odds of being diagnosed with migraine by approximately half. The greatest protective effect came from co-worker support (adjusted OR = 0.49, 95% CI: 0.39–0.60), followed by co-worker help (adjusted OR = 0.52, 95% CI: 0.42–0.64) and relationship with supervisors (adjusted OR = 0.52, 95% CI: 0.41–0.65).

## 4. Discussion

### 4.1. Findings of This Study

In a representative sample of bank employees, approximately 36% did not perceive sufficient social support in the workplace. We found that participants who reported low workplace social support were more likely to have migraine, which verified our hypothesis. Specifically, when compared to a low level of social support in the workplace, a high level of social support significantly decreased the risk of migraine by 74% after adjusting for socio-demographic confounders. The magnitude of this association is substantial and particularly enlightening. Among employees with low levels of social support in the workplace, the prevalence of migraine could be lowered from 43% to 13% if high levels of social support in the workplace were provided. All aspects of workplace social support have a protective function against migraine, but individuals benefit the most from co-worker support. Our findings point to the important role of workplace social support in migraine management and prevention.

Previous research has suggested that perceptions of social support may influence cardiovascular, neuroendocrine and immunological systems, hence reducing adverse health consequences [11,50]. Furthermore, social support is regarded as an encouragement to engage in healthy behaviours [51,52]. In an intervention study among patients with chronic pain, the intervention group who received a social support intervention had lower pain perception and pain interference than the control group who received a standard treatment [53].

The benefit of social support from the workplace for migraine has been well documented in Brazil [13,54] and Canada [55,56]. In Brazil, people who enjoyed high levels of social support in the workplace were 33% less likely to suffer from migraine [13]. In a Canadian study [56], no difference in migraine prevalence was observed between groups with high and low workplace social support, whereas Wilkins and Beaudet [55] detected a significant association between a high level of support from co-workers and a low likelihood of migraine, with an odds ratio of 0.67. This finding is consistent with ours, confirming the value of co-worker support in protecting against migraine.

However, it is worth noting that the beneficial effect of social support in the workplace on migraine is the strongest in the Chinese social context compared to other countries. Possible explanations could be as follows. Firstly, in China, most of the time is spent in the workplace, averaging 60.73 h per week, much above the International Labour Organisation standard of less than 40 h per week [57]. According to studies comparing social networks between Chinese and American workers [58] and between mainland Chinese and Hong Kong Chinese from different systems [59], workers in mainland China are found to have closer relationships with their colleagues and supervisors and are more likely to turn to them for support when needed. A further explanation is that China is a collective society that prioritises group goals over individual goals, encouraging interdependence and mutual collaboration [60]. Given this social context, in contrast to workers in other countries, Chinese workers tend to avoid conflict in the workplace and try to amend or improve relationships with others because they care about group goals [61].

A valid and reliable Chinese-language DCSQ Support scale is also presented in this study, which was applied to a sample of bank employees. The content and face validity of the Chinese version of the DCSQ Support scale are satisfactory in terms of the concept to be captured [46]. In previous works, explanatory factor analysis or principal component analysis was primarily used for structural validity [23,25]. However, the CFA used in this study is considered the “gold standard” technique for evaluating structural validity since it is hypothesis-driven, deductive and stringent in its approach [62]. We identified a clear one-dimensional factorial pattern of the Support scale that perfectly corroborated the original theoretical conceptual framework for evaluating social support in the workplace. The Cronbach’s α and item-total correlations are sufficient; hence, there is no reduction in the internal consistency of the scale when it is translated into Chinese, just as it was translated into Spanish [24], Japanese [25], German [23], English [23], and Norwegian [63]. Furthermore, the detailed examination of the weighted Kappa and percentage agreement for each item reveals excellent temporal stability, implying that this scale has the ability to remain consistent across time.

### 4.2. Strengths and limitations

As far as we know, this is the first study to examine the association between migraine and workplace social support in the social context of China. The following are the strengths of the present study. First, before applying the DCSQ Support scale to China, this study validated it cross-culturally. Our sample of bank employees fit the DCSQ’s target population, the employed population, whereas a review reported that recent validation studies typically relied on student samples and failed to justify the choice of the sample source [64]. The validity and reliability of the Support scale of the DCSQ were ensured in the Chinese context. Furthermore, probability sampling was used to select the sample, allowing statistical inferences to be made. Still, given the growing public health significance of migraine in recent years, the benefit of workplace social support for migraine merits more research to improve generalisability. Another contribution of our study is the application of the PMLE method to logistic regression. Sparse data are often found in prevalence studies [65], which means that an independent variable is fully separated by a single dependent variable. When employing the traditional maximum-likelihood estimation (MLE) method for logistic regression of sparse data, the model may produce bias; however, the PMLE method can solve this problem [66].

We must also point out the study’s limitations. The first is that the self-reported nature of the survey raised the possibility of biases, such as inaccurate recall or social-desirability reporting. Nevertheless, we believe the biases are small because the stability of the results has been verified by the test-retest reliability. Second, this investigation did not include more independent variables, which restricted the control for additional confounding and the analysis of interactions. Further research is required to gain more insight into the protective role of social support in the workplace. Additionally, the cross-sectional study design precluded any causal or temporal conclusions about how workplace social support influenced migraine. Future longitudinal studies are warranted.

### 4.3. Implications

Our results highlight the protective value of workplace social support against migraine in the social context of China. This work underpins the design and implementation of workplace intervention programmes aimed at boosting social support and fostering positive health outcomes. Interventions targeted at high-risk populations are crucial, since universal interventions, especially in a country with a large population like China, can be ineffective [67]. Better management and prevention of migraine would reduce the enormous health burden associated with migraine and increase the productivity of employees.

This study also gives researchers and practitioners a valid and reliable scale to measure workplace social support in China. Although the Job Content Questionnaire (JCQ) already exists to measure psychosocial exposures in occupational health research, the DCSQ is reported to be more objective and practical [25,63]. On one hand, the DCSQ is able to perceive the workplace atmosphere in addition to what the JCQ observes [68]; while on the other, the DCSQ is shorter, enabling easier use [25].

## 5. Conclusions

Our findings demonstrate the protective effect of workplace social support on migraine in the Chinese social context, with the strongest benefit coming from co-workers. Further research is needed to include more variables to explore interactive effects and potential confounding to gain a deeper understanding of the protective role of social support in the workplace. This study also provides a Chinese version of the DCSQ Support scale as a valid and reliable instrument for measuring workplace social support in China.

## Figures and Tables

**Table 1:** Socio-demographic characteristics.

Characteristics	All Participants (N = 677)	Positive for Migraine (N = 160)	Negative for Migraine (N = 517)	Chi-Square *p*-Value
	N (%) *	N (%) *	N (%) *	
Age (years)				0.000
18–29	148 (21.86)	0 (0)	148 (100)	
30–39	173 (25.56)	43 (24.86)	130 (75.14)	
40–49	195 (28.80)	105 (53.85)	90 (46.15)	
50–60	161 (23.78)	12 (7.45)	149 (92.55)	
Sex				0.000
Male	258 (38.07)	29 (11.24)	229 (88.76)	
Female	419 (61.93)	131 (31.26)	288 (68.74)	
Education				0.000
College or below	120 (17.73)	9 (7.50)	111 (92.50)	
University	453 (66.91)	103 (22.74)	350 (77.26)	
Graduate or above	104 (15.36)	48 (46.15)	56 (53.85)	
Occupation				0.000
Administration and general affairs	143 (21.12)	12 (8.39)	131 (91.61)	0.000
Marketing and loan services	287 (42.39)	104 (36.24)	183 (63.76)	
Cash operation	205 (30.28)	44 (21.46)	161 (78.54)	
Professionals	42 (6.21)	0 (0)	42 (100)	
Location of work				0.202
Urban	648 (95.72)	156 (24.07)	492 (75.93)	
Rural	29 (4.28)	4 (13.79)	25 (86.21)	
Relationship status				0.003
Married or in a relationship	511 (75.48)	135 (26.42)	376 (73.58)	
Not in a relationship	166 (24.52)	25 (15.06)	141 (84.94)	
Monthly salary (¥)				0.006
Under 8000	352 (52.00)	100 (28.41)	252 (71.59)	
8001–11,000	206 (30.43)	42 (20.39)	164 (79.61)	
11,001–14,000	68 (10.04)	15 (22.06)	53 (77.94)	
Above 14,001	51 (7.53)	4 (7.84)	47 (92.16)	
Social support in the workplace				0.000
Low	107 (15.80)	46 (42.99)	61 (57.01)	
Medium	137 (20.24)	56 (40.88)	81 (59.12)	
High	433 (63.96)	58 (13.39)	375 (86.61)	

* Weighted number and percentage.

**Table 2:** Weight Kappa and percentage agreement for each item of the DCSQ Support scale.

No	Item	Weighted Kappa (95% CI)	%Agreement
1	Atmosphere	0.81 (0.77–0.86)	85.23%
2	Spirit of unity	0.83 (0.79–0.87)	86.71%
3	Co-worker support	0.83 (0.79–0.87)	86.26%
4	Co-worker help	0.87 (0.83–0.90)	88.92%
5	Relationship with supervisors	0.83 (0.79–0.87)	85.82%
6	Relationship with co-workers	0.83 (0.78–0.87)	86.41%

**Table 3:** Associations between social support in the workplace and migraine diagnosis.

Dependent Variable		Crude OR (95% CI)	Adjusted OR (95% CI) ^#^
Total workplace social support	High	0.20 (0.13–0.33) ***	0.26 (0.14–0.46) ***
	Medium	0.91(0.55–1.52)	0.85 (0.45–1.62)
	Low	Reference	Reference
Aspects	Atmosphere (1 to 4 score)	0.57 (0.47–0.69) ***	0.54 (0.42–0.70) ***
	Spirit of unity (1 to 4 score)	0.63 (0.52–0.75) ***	0.58 (0.46–0.73) ***
	Co-worker support (1 to 4 score)	0.49 (0.41–0.58) ***	0.49 (0.39–0.60) ***
	Co-worker help (1 to 4 score)	0.51 (0.43–0.60) ***	0.52 (0.42–0.64) ***
	Relationship with supervisors (1 to 4 score)	0.56 (0.47–0.67) ***	0.52 (0.41–0.65) ***
	Relationship with co-workers (1 to 4 score)	0.49 (0.40–0.59) ***	0.53 (0.42–0.68) ***

^#^ Adjusted for gender, age, education, relationship, location of work, job and salary *** *p* < 0.001.

## Data Availability

Not applicable.

## Supplementary Materials

The following supporting information can be downloaded at: https://www.mdpi.com/article/10.3390/healthcare11020171/s1, Figure S1: Translation process of the Support scale of DCSQ; Table S1: Descriptive statistics of the six items of the Support scale of DCSQ.
