# Supplementary material for: Association between Migraine and Workplace Social Support in the Social Context of China: Using a Validated Chinese Version of the DCSQ
Source: Healthcare (Basel). 2023 Jan 5;11(2):171. doi: 10.3390/healthcare11020171 (PMC9859248; doi:10.3390/healthcare11020171)
Supplement: Supplementary file 1 [file healthcare-11-00171-s001.zip › Supplementary Files/Table S1.pdf]

Table S1. Descriptive statistics of the six items of the Support scale of DCSQ.

No.	Item	Median (Range)	Skewness	Kurtosis
(1)	There is a cohesive atmosphere where I work.	4 (1 to 4)	−1.18	0.28
(2)	There is a good spirit of unity.	4 (1 to 4)	−1.12	0.13
(3)	My co-workers support me.	4 (1 to 4)	−1.01	−0.37
(4)	The others are ready to hear my demands.	4 (1 to 4)	−0.96	−0.43
(5)	I get on well with my supervisors.	4 (1 to 4)	−0.90	−0.42
(6)	I get on well with my co-workers.	4 (1 to 4)	−1.13	−0.05
Multivariate normality			78.9015 ***	274.0762 ***

*** $p < 0.001$.
