# Supplementary figures and images for: Association between Migraine and Workplace Social Support in the Social Context of China: Using a Validated Chinese Version of the DCSQ
Source: Healthcare (Basel). 2023 Jan 5;11(2):171. doi: 10.3390/healthcare11020171 (PMC9859248; doi:10.3390/healthcare11020171)

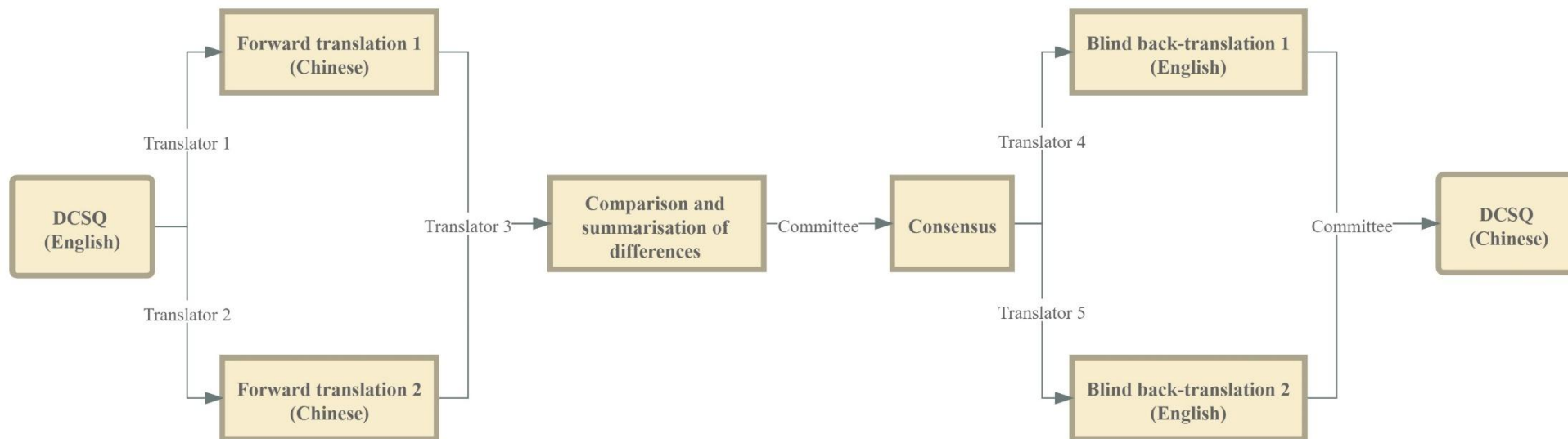

Supplement: Supplementary file 1 [file healthcare-11-00171-s001.zip › Supplementary Files/Figure S1.pdf]
